# Chronotype’s effect on academic achievement and absence from classrooms and clinical sessions among clinical phase medical students at the Faculty of Medicine, University of Tabuk, Saudi Arabia

**DOI:** 10.3389/fpsyg.2025.1664598

**Published:** 2025-12-10

**Authors:** Sawsan Mohammed Alblewi, Hyder Mirghani

**Affiliations:** Faculty of Medicine, University of Tabuk, Tabuk, Saudi Arabia

**Keywords:** chronotype, medical students, absence, academic achievements, Saudi Arabia

## Abstract

**Objectives:**

Chronotype refers to an individual’s unique biological clock determined by bedtime preferences and daytime activities. The contradiction between sleep preferences and daily routines (circadian misalignment) could affect academic performance among students. This important issue has been a topic of considerable debate. We aimed to assess the chronotype’s effect on academic achievement and absence from classrooms and clinical sessions among medical students at the University of Tabuk.

**Methods:**

A cross-sectional study was conducted among 224 medical students at the University of Tabuk, Saudi Arabia, from March 2024 to August 2024. A structured Web-based questionnaire was designed based on the Morningness-Eveningness Questionnaire (MEQ), age, absence from classrooms and clinical sessions, and the cumulative grade average (GPA). Data analysis was conducted using the Statistical Package of the Social Science Software (SPSS), version 20, New York.

**Results:**

Out of the 224 medical students (age 23.29 ± 1.87 years), 61.2% were categorized as having an intermediate chronotype, while 23.2% were identified as moderate evening chronotypes. Moderate morning and definite evening chronotypes were reported in 11.2 and 4.5%, respectively. A negative correlation was found between classroom absenteeism and GPA (95% *CI*, 0.053–0.749, *p*-value, 0.017). A positive correlation was found between students’ age and GPA (95% *CI*, 1.308–1.971, *p*-value, 0.000). No association was evident between the GPA, time of the study (95% *CI*, 0.648–3.660, *p*-value, 0.0329), and chronotype (95% *CI*, 0.931–1.004, *p*-value, 0.079).

**Conclusion:**

Medical students at the University of Tabuk in Saudi Arabia were predominantly identified as intermediate and evening chronotypes. The chronotype was associated with classroom absenteeism and GPA. Further multicenter studies investigating the determinants of chronotypes are recommended.

## 1 Introduction

Many biological rhythms in the human body occur in cyclic patterns, with the circadian rhythm being the most common. The sleep/wake cycle is a typical example that lasts over 24 h. The central circadian clock is located in the suprachiasmatic nucleus of the hypothalamus and receives light and darkness signals from the retina to correlate them in accordance with sleep time preferences (Montaruli et al., 2021; Gębska et al., 2022). A unique personal biological clock, known as a chronotype, is determined by bedtime preferences and daytime activities and is largely determined by internal biological factors (Park et al., 2018; Adan et al. 2012). Although circadian preferences exist on a continuum, they can be divided into morning, evening, and intermediate types. The evening and morning chronotypes are further divided into definite and intermediate categories. A sharp shift toward the evening chronotype is observed during adolescence, reaching its peak in early youth, followed by a steady change toward the morning chronotype as individuals age (Barclay et al., 2016).

Sleep is necessary for several key bodily processes, including supporting memory, modulating the immune response, and removing waste from the brain. Sleep disorders are common among university students, affecting their quantity, quality, and regularity of sleep, and these issues can negatively impact their academic performance (Said and Miyamoto, 2025; Suardiaz-Muro et al., 2020). Sleep patterns, which comprise not only the quantity and quality of sleep but also the timing of sleep in relation to natural sleep cycles, known as chronotypes, have been connected to academic achievement and learning outcomes (Barley et al., 2023). Chronotypes (the innate natural sleep–wake pattern) are divided into three types: morning, evening, and intermediate, with 40% of the adult population belonging to the morning/evening category and 60% belonging to the intermediate category (Reddy and Nagothu, 2019).

Medical students are crucial part of the community with high academic demands and busy schedules. Importantly, the academic performance of evening chronotype students in comparison to their morning chronotype counterparts can be negatively impacted by university class schedules that contradict their circadian inclinations (circadian misalignment). On the other hand, morning chronotype students may have an advantage in their mental performance because they are more in line with their daily routine (Lovibond and Lovibond, 1995). Evening chronotype students have irregular sleep schedules and increased daytime sleepiness. Therefore, they are more likely to oversleep, take daytime naps, and miss early morning (Arastoo et al., 2024). The association between classroom attendance and academic performance is a subject of controversy. Ta et al. (2020) found that poor attendance is associated with poor academic achievement, while Laird-Fick et al. (2018) found no such association. The effects of chronotypes on academic performance is also debated, with some studies finding an association (Akram et al., 2018; Gupta et al., 2023), while others reporting no relationship (Shahid et al., 2012; Calculator.net, 2025). We hypothesize that evening chronotypes are more prone to daytime sleepiness and, therefore, may have higher absenteeism in classrooms due to circadian misalignment (as they tend to perform better during the evening). Since examinations are usually held in the morning, we hypothesize that evening chronotypes may perform poorly on these assessments. The existing literature on the association between chronotype, academic performance, and absence from classroom is scarce. Therefore, this study aimed to assess the effects of chronotypes on academic achievement and absence from classrooms and clinical sessions among clinical phase medical students at the Faculty of Medicine, University of Tabuk.

## 2 Methods

**Figure 1:**
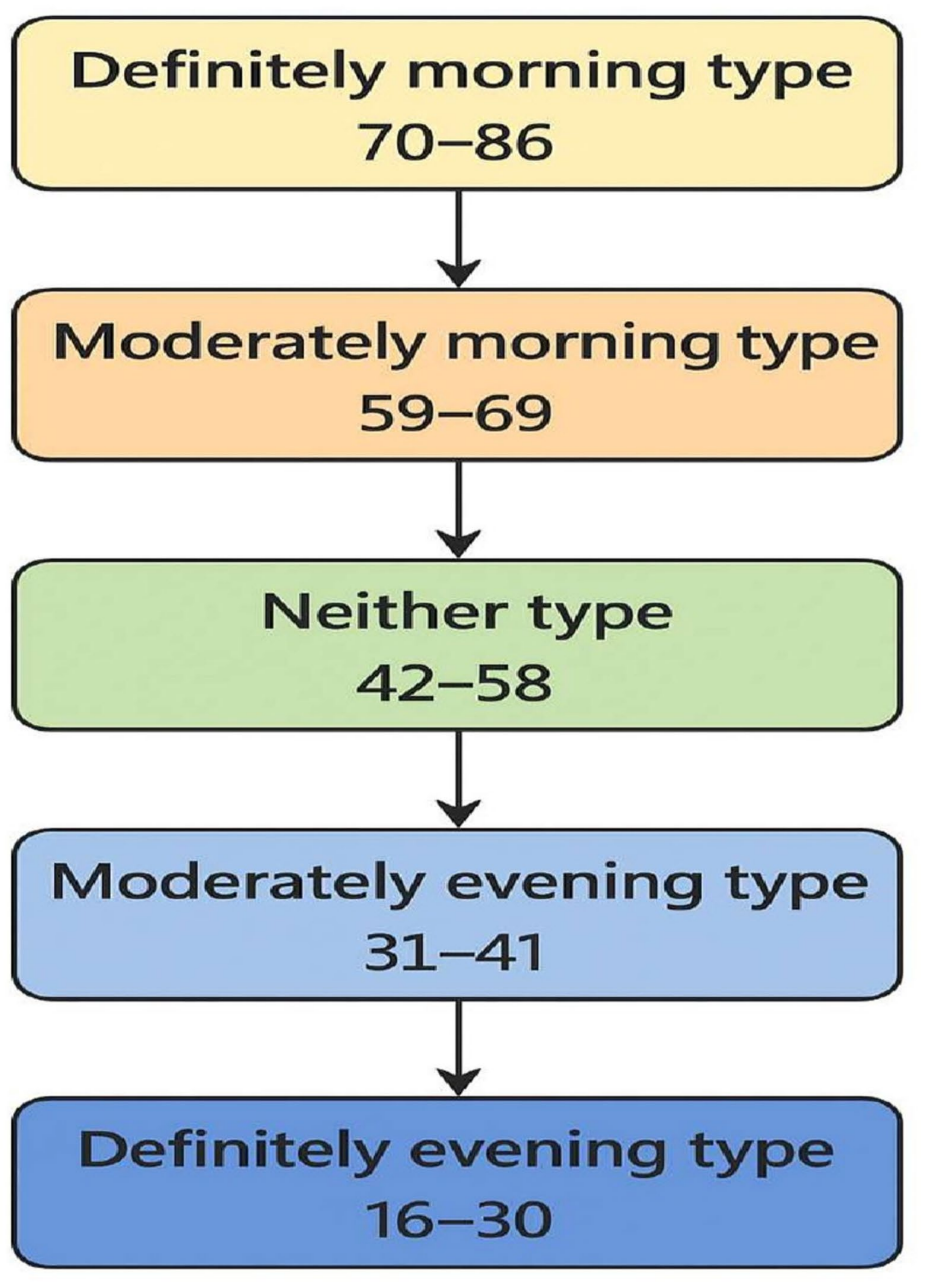
Chronotype classification flowchart.

A cross-sectional study was conducted among 224 clinical phase medical students at the Medical College, University of Tabuk, Saudi Arabia, from March 2024 to August 2024. A structured Web-based questionnaire was designed based on the Morningness-Eveningness Questionnaire (MEQ), age, absence from classrooms and clinical sessions, and the cumulative grade average (GPA). The maximum score for the cumulative grade average is 5 points, with a score of ≥3.7 points considered good, while a score < 3.7 points considered average. The Morningness-Eveningness Questionnaire has been previously validated for use among university students (Shahid et al., 2012). This questionnaire consists of 19 items; each section of the scale is assigned a value of 1 to 5 points, with a maximum score of 95 points. To obtain a global score, each item is calculated and the sum is then converted to a 5-point scale. Based on the total score (calculated from the sum of 19 questions), the chronotype was divided into the following categories: definitely morning type, 70–86 total points; moderately morning type, 59–69 total points; neither type, 42–58 total points; moderately evening type, 31–41 total points, and evening type, 16–30 total points (Figure 1).

The previous cumulative grade average (CGA) and the number of absences per month were collected from the Vice Deanship of Academic Affairs after obtaining the student’s consent. The student’s absence was reported as a total number of absences per month: 1–3/month, 4–6/month, and 7–10/month.

### 2.1 Sampling technique

This study employed a simple random technique.

### 2.2 Inclusion criteria

This study included medical students who were in the clinical phase of their studies at the Faculty of Medicine, University of Tabuk.

### 2.3 Exclusion criteria

This study excluded students who either refused to participate in the questionnaire or were not in the clinical phase.

### 2.4 Sample size calculation

The sample size was calculated from the total number of clinical phase students (*n* = 465), using an online calculator; the estimated sample size was 211, with a 95% confidence level. The link to the sample size calculator is as follows: https://www.calculator.net/sample-size-calculator.html?type=1&cl=95&ci=5&pp=50&ps=465&x=Calculate (Zhang et al., 2018).

### 2.5 Outcome measures

The outcome measures include the following:

The effects of chronotypes on academic achievement among clinical phase medical students at the Faculty of medicine, University of Tabuk, Saudi Arabia.
The effects of absence from classrooms and clinical sessions on academic performance among clinical phase medical students at the Faculty of medicine, University of Tabuk, Saudi Arabia.

### 2.6 Ethical issues

Ethical approval was obtained from the Research Ethical Committee at the University of Tabuk (UT-352-183-2024). Participants were informed that their participation is voluntary and that filling out the questionnaire indicates their consent to participate.

### 2.7 Non-response rate and incomplete responses

In this study, the non-response rate was identified by comparing the total number of students invited to participate with the number of students who completed the questionnaire. Incomplete responses were identified through a preliminary data screening process; questionnaires with missing key variables (such as chronotype score or GPA) were excluded from the final analysis.

### 2.8 Statistical analysis

Data were entered into a computer using Microsoft Office Excel (2016) for Windows. The data were then transferred to the Statistical Package of the Social Science Software (SPSS) program, version 20 (IBM SPSS Statistics for Windows, Version 20.0. Armonk, NY, IBM Corp.) for statistical analyses. The data were presented as percentages and mean± SD, and the binary logistic regression analysis was performed to assess the association between chronotypes, absence from classrooms/clinical sessions, and academic performance. A *p*-value of < 0.05 was considered statistically significant.

## 3 Results

In this study, we included 224 clinical phase medical students who were aged between 19 and 30 years (mean± SD, 23.29 ± 1.87 years). The cumulative grade average ranged from 2.3 to 5 (mean± SD, 4.06 ± 0.63). Good grades were observed in 72.8% of the students and average grades were reported by 27.2%. The absence from classrooms was 1–3 days/month in 68.8% of the students, 4–6 days/month in 25%, and 7–10 days/month in 6.3%. The majority of students (75.9%) preferred morning classes (Table 1).

**Table 1:** Age, cumulative grade average, absence from classrooms, and time preference for lectures and clinical sessions.

Variable	Mean± SD
Age/years (19–30)	23.29 ± 1.87
Cumulative grade average (2.3–5)	4.06 ± 0.63
Good	163(72.8%)
Average	061(27.2%)
Variable no% (total number = 224)	
Absence from classroom	
1–3/month	154 (68.8%)
4–6/month	056 (25.0%)
7–10/month	014 (6.3%)
Time preference for lectures and clinical sessions	
Morning	170 (75.9%)
Afternoon	054 (24.8%)

Approximately two-thirds of the students (61.2%) had an intermediate chronotype, followed by moderate evening (23.2%) and moderate morning (11.2%) chronotypes. Importantly, 4.5% of the students reported a definite evening chronotype, whereas none reported a definite morning chronotype (Table 2).

**Table 2:** Chronotypes among clinical phase medical students at the University of Tabuk, Saudi Arabia.

Chronotype	No% (total number = 224)
Definitely morning	0.0 (0%)
Moderately morning	25 (11.2%)
Intermediate	137 (61.2%)
Moderately evening	52 (23.2%)
Definitely evening	10 (4.5%)

The morningness/eveningness questionnaire yielded the following results: 3.44 ± 0.96 for choosing 5 consecutive hours to work, 3.13 ± 1.01 for determining when your “feeling best” peak, and 3.53 ± 1.92 for determining whether you consider yourself a morning or evening person. The lowest scores were as follows: 1.94 ± 1.055 for the extent to which you depend on being woken by an alarm clock, 1.8 ± 0.85 for how hungry you feel within the first half-hour after waking up in the morning, and 1.65 ± 0.477 for which of the four testing times you would choose for a better performance. The different components of the morningness/eveningness questionnaire are depicted in Table 3.

**Table 3:** Morningness/Eveningness Questionnaire components.

Number	Question	Mean± SD
1	What time would you get up if you were entirely free to plan your day?	2.57 ± 1.63
2	What time would you go to bed if you were entirely free to plan your evening?	2.24 ± 1.31
3	If there is a specific time at which you have to get up in the morning, to what extent do you depend on being woken up by an alarm clock?	1.94 ± 1.05
4	How easy do you find it to get up in the morning (when you are not woken up unexpectedly)?	2.42 ± 0.87
5	How alert do you feel during the first half hour after you wake up in the morning?	2.49 ± 0.86
6	How hungry do you feel during the first half-hour after you wake up in the morning?	1.80 ± 0.85
7	During the first half-hour after you wake up in the morning, how tired do you feel?	2.30 ± 0.86
8	If you have no commitments the next day, what time would you go to bed compared to your usual bedtime?	2.13 ± 1.01
9	You have decided to engage in some physical exercise. A friend suggested that you do this for one hour twice a week, and the best time for him is between 7:00 and 8:00 a.m. Bearing in mind nothing but your own internal “clock,” how do you think you would perform?	2.56 ± 0.92
10	At what time of the day do you feel that you become tired as a result of the need for sleep?	3.00 ± 1.31
11	You want to be at your peak performance for a test that you know is going to be mentally exhausting and will last 2 h. You are entirely free to plan your day. Considering only your own internal “clock,” which one of the four testing times would you choose?	1.65 ± 0.48
12	If you got into bed at 11:00 p.m., how tired would you be?	2.21 ± 1.32
13	For some reason, you went to bed several hours later than usual, but there is no need to get up at any particular time the next morning. Which one of the following are you most likely to do?	2.18 ± 1.01
14	One night, you have to remain awake between 4:00 and 6:00 a.m. to carry out a night watch. You have no commitments the next day. Which one of the alternatives suits you best?	2.55 ± 1.12
15	You have to do 2 h of hard physical work. You are entirely free to plan your day and consider only your own internal “clock.” Which one of the following times would you choose?	2.08 ± 0.90
16	You have decided to engage in hard physical exercise. A friend suggests that you do this for one hour twice a week, and the best time for him is between 10:00 and 11:00 p.m. Bearing in mind nothing else but your own internal “clock,” how well do you think you would perform?	2.08 ± 0.90
17	Suppose that you can choose your own work hours. Assume that you worked a 5-h day (including breaks) and that your job was interesting and paid for results. Which five consecutive hours would you select?	3.44 ± 0.96
18	At what time of the day do you think you reach your “feeling best” peak?	3.13 ± 1.10
19	One hears about “morning” and “evening” types of people. Which one of these types do you consider yourself to be?	3.54 ± 1.92

In this study, a negative correlation was found between absence from classrooms and cumulative grade average [SE, −1.913, B, −1.61, Exp (B), 0.22, 95% *CI*, 0.053–0.749, and *p*-value, 0.017], with significant statistical difference. A positive correlation was found between students’ age and cumulative grade average [SE, 0.528, B, 0.474, Exp (B), 1.606, 95% *CI*, 1.308–1.971, and *p*-value, 0.000], with significant statistical difference. However, no significant statistical difference was evident between the cumulative grade average, the time of study [SE, 0.531, B, 0.43, Exp (B), 0.329, 95% *CI*, 0.648–3.660, and *p*-value, 0.0329], and the chronotype [SE, 3.56, B, −0.034, Exp (B), 0.967, 95% *CI*, 0.931–1.004, and *p*-value, 0.079] (Table 4).

**Table 4:** Correlation between cumulative grade average, absence from classrooms, age, and study time preference.

Character	SE	B	Exp (B)	95% *CI*	*P*-value
Absence from classrooms	−1.91	−1.61	0.20	0.05–0.75	0.017^*^
Study preference	0.53	0.43	0.33	0.65–3.66	0.329
Chronotype	3.56	−0.03	0.97	0.93–1.00	0.079
Age	0.53	0.47	1.61	1.31–1.97	0.000^**^

^*^Significant.

^**^Highly significant.

## 4 Discussion

In the current study, the majority of medical students were classified as the intermediate chronotype, followed by the moderate evening chronotype. The findings were in line with a study conducted on college students in China, which found intermediate and evening chronotypes in 51.17 and 45.14%, respectively (Tong et al., 2023). Zhang et al. (2018) conducted a study among medical students in China and reported that the majority of students are intermediate (62.8%), followed by definite morningness (22.9%), and definite eveningness (14.3%). The findings reported by Zhang et al. (2018) are similar to the current results, in which 61.2% were of intermediate chronotype. However, the current data showed a higher prevalence of eveningness chronotype, at 27.7%, compared to the findings reported by Zhang et al. (2018).

An interesting study found a shifting pattern of chronotypes, with some students switching from a moderate evening chronotype to a morning chronotype and others shifting from morning to evening chronotypes over time. The authors attributed these changes to coffee consumption after 5:00 p.m. (Barley et al., 2023). Other factors that influence circadian preferences are attention-deficit/hyperactivity disorder (Becker et al., 2024) and depression symptomatology (Porcheret et al., 2018). Importantly, living farther from the equator, spending more time outdoors, and experiencing later sunsets are all associated with the evening chronotype (Van den Berg et al., 2018).

Sleep disorders, such as social jetlag (circadian misalignment), are common among medical students due to their busy schedules, high academic demands, and prolonged periods of intensive study (Azad et al., 2015).

Sato et al. (2024) found lower levels of learning motivation in evening chronotypes and suggested scheduling the most important course topics in the afternoon to enhance learning and academic performance. A recent study (Niroumand Sarvandani et al., 2025) conducted in Iran found that circadian misalignment is associated with boredom and poor mental status, including depression, anxiety, and suicidal ideation, which could negatively impact their academic performance. The chronotype effects on learning are not universal. Reddy and Nagothu (2019) conducted a study in India and found that chronotypes could influence fluid intelligence rather than crystallized intelligence. Therefore, practical learning could be influenced more by circadian misalignment. A recent study from Asia (Manjareeka et al., 2025) found that individuals with an evening chronotype experience higher levels of stress and demonstrate poorer academic performance. The study also suggested that improving sleep hygiene could help reduce psychological stress and enhance academic achievement.

The association between chronotypes and academic performance is complex and multifactorial. In this study, no association was found between chronotypes and cumulative grade average [B, −0.034, Exp (B), 0.967, 95% *CI*, 0.931–1.004, and *p*-value, 0.079]. Our findings were in line with the findings reported by Balcı and Çalışkan (2022), who conducted a multi-center study among university students in Turkey and found no association between chronotypes and academic achievement. The authors found higher grades among students who adopted the kinesthetic learning style compared to their counterparts with auditory and visual learning styles. The current findings support the findings of a previous study conducted in Saudi Arabia, which found no association between the chronotype and cumulative grade average (Mirghani et al., 2019). A study conducted among medical students in Sudan (Mirghani, 2017) found lower academic achievement among evening chronotypes. However, the authors used the mid-sleep time and not the morningness/eveningness questionnaire.

In this study, a negative correlation was found between absence from classrooms and cumulative grade average [B, −1.61, Exp (B), 0.22, 95% *CI*, 0.053–0.749, and *p*-value, 0.017], with a significant statistical difference. A previous study conducted in Tabuk, Saudi Arabia, found no association between absence from classrooms, chronotypes, and cumulative grade average, in contrast to the present findings (Aqahtani et al., 2023). Hakimi et al. (2024) conducted a study among nursing students in Iran and found high burnout among evening chronotypes in terms of professional efficacy, emotional exhaustion, and cynicism. Students with early-start schedules, particularly those who are evening chronotypes, are at a higher risk of sleep debt, daytime sleepiness, and reduced alertness during morning classes, which can translate into higher rates of lateness and absenteeism. A study found that academic performance improves when class schedules align with students’ biological clocks (morning chronotypes perform better in the morning sessions, while evening chronotypes are better in the afternoons). Therefore, school start time alignment with the circadian clock is vital for better academic performance (Goldin et al., 2020; Rodríguez Ferrante et al., 2023).

The connection between poor academic performance and chronotypes may be mediated by factors such as poor sleep quality and a lower quality of life experienced by evening chronotypes (Balcı and Çalışkan, 2022). Mobile phone dependence can further contribute to poor academic achievement, as it negatively impacts health-related quality of life. Ying et al. (2024) found that mobile phone dependence significantly predicted chronotypes, and chronotypes negatively influenced the health-related quality of life among college students. An interesting study conducted among medical students in China found a link between late chronotype, sedentary lifestyle, and caffeinated drink consumption, indicating that exercise and caffeinated drink reduction could be effective interventional strategies (Zhang et al., 2018).

The limitations of the study include reliance on a self-reported questionnaire, the fact that it was conducted at a single college, and the focus was on clinical-phase students, restricting the generalizability of the findings.

## 5 Conclusion

Medical students at the University of Tabuk in Saudi Arabia were mostly intermediate and evening chronotypes. CGA was associated with the evening chronotype, age, and absence from classrooms. No association was found between CGA and study preference. Further multicenter studies investigating the determinants of chronotypes are recommended.

## Data Availability

The original contributions presented in the study are included in the article/supplementary material, further inquiries can be directed to the corresponding author/s.
